# A System-Agnostic, Adaptable and Extensible Animal Support Cradle System for Cardio-Respiratory-Synchronised, and Other, Multi-Modal Imaging of Small Animals

**DOI:** 10.3390/tomography7010004

**Published:** 2021-02-07

**Authors:** Veerle Kersemans, Stuart Gilchrist, Philip Danny Allen, Sheena Wallington, Paul Kinchesh, John Prentice, Martin Tweedie, Jamie H. Warner, Sean C. Smart

**Affiliations:** 1 Preclinical Imaging Scientific Research Facility, Department of Oncology, University of Oxford, Oxford OX3 7DQ, UK; stuart.gilchrist@oncology.ox.ac.uk (S.G.); pda796@gmail.com (P.D.A.); s.wallington@griffininstitute.org.uk (S.W.); paul.kinchesh@oncology.ox.ac.uk (P.K.); john.prentice@oncology.ox.ac.uk (J.P.); sean.smart.imaging@gmail.com (S.C.S.); 2 Department of Materials, Quantum Information Processing Interdisciplinary Research Collaboration, University of Oxford, Parks Road, Oxford OX1 3PH, UK; mtweedie92@gmail.com (M.T.); jamie.warner@austin.utexas.edu (J.H.W.)

**Keywords:** imaging-compatible, multi-modal imaging, multi-vendor, handling apparatus, optimisation

## Abstract

Standardisation of animal handling procedures for a wide range of preclinical imaging scanners will improve imaging performance and reproducibility of scientific data. Whilst there has been significant effort in defining how well scanners should operate and how in vivo experimentation should be practised, there is little detail on how to achieve optimal scanner performance with best practices in animal welfare. Here, we describe a system-agnostic, adaptable and extensible animal support cradle system for cardio-respiratory-synchronised, and other, multi-modal imaging of small animals. The animal support cradle can be adapted on a per application basis and features integrated tubing for anaesthetic and tracer delivery, an electrically driven rectal temperature maintenance system and respiratory and cardiac monitoring. Through a combination of careful material and device selection, we have described an approach that allows animals to be transferred whilst under general anaesthesia between any of the tomographic scanners we currently or have previously operated. The set-up is minimally invasive, cheap and easy to implement and for multi-modal, multi-vendor imaging of small animals.

## 1. Introduction

Preclinical imaging is established as an integral part of biomedical research and the associated development pipeline for new biomarkers, drugs and therapies [1,2,3]. Whilst much effort has been focussed on optimisation of contrast agents, hardware design and image reconstruction, much less attention has been paid to the animal handling and physiological monitoring apparatus. Although many preclinical imaging systems are supplied with the basic apparatus for animal restraint, physiological monitoring and generation of cardio-respiratory gating/triggering signals, most of these apparatuses are not comprehensive and are incompatible across vendors where multi-modal imaging is required [4,5].

Many small-animal imaging and image-guided radiotherapy procedures require the use of general anaesthesia primarily for immobilisation and for minimising stress related to the insertion of invasive devices. Whatever the reason, they all necessitate reliable means to position the animal within the scanner or radiotherapy device, monitor the animal’s vital signs, assess anaesthetic depth and provide thermoregulation in a manner that is compatible with the imaging and/or radiotherapy systems being used. Changes in the cardio-respiratory rate, pattern and amplitude can be monitored, and although some experience is needed to interpret their significance, they can be indicators of anaesthetic-related physiological distress as well as underlying disease. In addition to the animal welfare considerations, cardio-respiratory monitoring also allows for cardio-respiratory gating/triggering to synchronise the imaging to their respective cycles and avoid motion-related image degradation [6,7,8].

Consequently, there is an opportunity for optimisation and standardisation of the animal handling and monitoring apparatus across scanner modalities and manufacturers. Successful deployment will help improve the quality of physiological maintenance and animal welfare, maximise image quality, increase statistical power and also lower the barriers to imaging and expand the research capabilities of the preclinical imaging techniques [2]. However, certain requirements will need to be fulfilled to make this optimisation successful and generally applicable. Such multi-modal, multi-vendor compatible animal handling apparatus should be simple and cheap to manufacture, non- or minimally invasive and compact enough to be used in small confines, as dictated by the small imaging fields of, for example, magnetic resonance imaging (MRI) coils, positron emission tomography (PET) detectors or single-photon emission computed tomography (SPECT) collimators.

We have designed and implemented a multi-modal and multi-vendor compatible animal handling apparatus that is used in conjunction with a custom-made physiological signal processing unit; the latter allows cardiac- and respiratory-synchronised imaging to be performed on animals that are moved, in the cradle, between scanners. The animal support cradle can be adapted on a per application basis and features integrated tubing for anaesthetic and tracer delivery, an electrically driven rectal temperature maintenance system and respiratory and cardiac monitoring; all are PET-, SPECT-, computed tomography (CT)- and MRI-compatible and largely insensitive to false activations arising from local environment changes such as doors opening when using a pressure transducer for respiratory monitoring. The associated gating/triggering control signal generation is user-programmable and can be deployed on any system capable of using transistor–transistor logic (TTL) (or similar) gating control signals.

## 2. Methodology

### 2.1. Ethics Statement

All animal studies were performed in full compliance with national legislation and with the approval of the Oxford University Animal Welfare and Ethical Review Body.

### 2.2. Animals

Five male 8-week-old B6SJLCD45-WT mice (20.0–21.9 g; animals were bred locally) were housed in individual ventilated cages at constant temperature and humidity, and water and food were freely available. Anaesthesia was induced and maintained using isoflurane (1–4%) in air/oxygen (*v*/*v* 80/20); the animals recovered afterwards with no ill effects. Induction and recovery were performed in a heated unit with a base temperature of ~37 °C. Mice were placed prone into a flat-based multi-modal animal support cradle with a built-in carbon-fibre-sheet resistive heater, and the rectal temperature was set at 36.0 °C using a home-built heater driver [9]. The depth of anaesthesia was monitored using a novel graphene-based piezo-electric system measuring the animal’s respiration rate, which was maintained at 60–90 breaths/min. Cardiac and respiratory signals were processed for prospective gating on the MRI scanner and retrospective gating on the PET, SPECT and CT scanners, enabling production of cardio-respiratory-synchronised MRI, CT, PET and SPECT images.

### 2.3. Design of the Animal Handling Apparatus

All images and technical drawings with instructions for the 3D-printed components can be found at the Oxford University Research Archive at https://ora.ox.ac.uk/objects/uuid:4ad2c11e-2cda-4e2e-b79c-8c7d31577101 with the DOI:10.5287/bodleian:xqOX58rrY (resolving to https://doi.org/10.5287/bodleian:xqOX58rrY).

### 2.4. MRI System: Coil and Cradle Loading Apparatus

**Figure 1:**
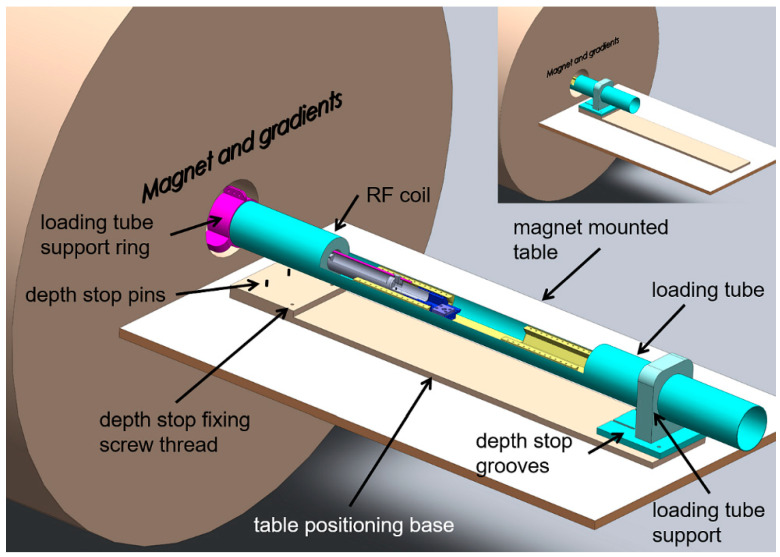
Overview schematic of the loading tube that contains the radiofrequency (RF) coil, hexbase adaptors and flat-base insert and that mounts to the table using a pin and groove-locating system. The animal cradle is shown positioned within the RF coil for imaging. The loading apparatus is centred in the magnet axially by the loading tube support ring and longitudinally by the depth stop pins and grooves formed on the magnet table and loading tube base, respectively. Figure 1 insert: the loading apparatus in position for imaging.

Our group operates MRI scanners that use magnets produced by Varian with electronic controls provided by both Varian (7.0 T) and Bruker (4.7 T). Each magnet features a built-in table at the front of and below the magnet bore. For these systems, we typically, though not exclusively, operate a coil-in-cradle loading apparatus, which positions the isocentre of the radiofrequency (RF) coil at the isocentre of both the magnet and the gradient set, as shown in Figure 1. This loading tube assembly consists of a combination of permanently installed and removable components.

**Figure 2:**
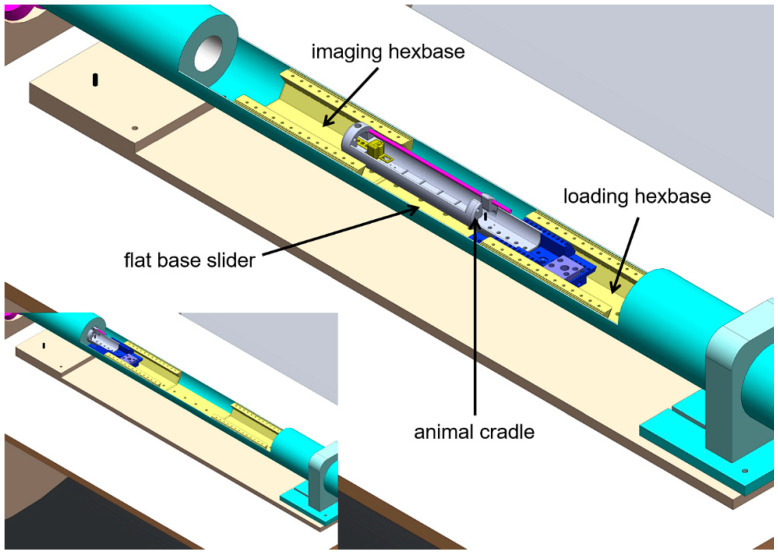
Schematic of the contents of the loading tube with the animal cradle located in the loading position and, Figure 2 insert, in the imaging position.

Within the loading tube, there are two hexagonally shaped dovetail (hexbase) adaptors (one is used during scanning, and the other during animal and cradle loading and unloading) and a flat-base insert in between the two that provides a constant plinth height for sliding the animal cradle between hexbase adaptors, as shown in Figure 2. The animal cradle base has a hexagonal profile that fits the hexbase adaptor and a vertically oriented screw is used to lift the animal cradle within the hexbase adaptor and lock the two together.

### 2.5. PET/SPECT/CT System: Gantry-to-Cradle Adaptor

**Figure 3:**
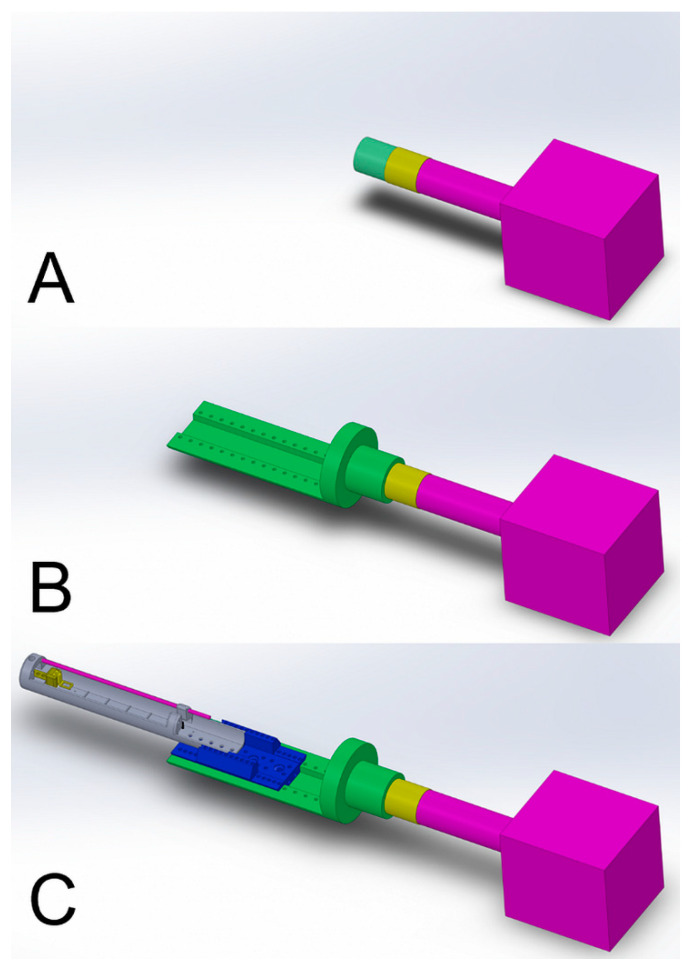
Schematic views of the cradle coupling unit on the positron emission tomography/single-photon emission computed tomography/computed tomography (PET/SPECT/CT) scanner. (**A**) The purple block represents the gantry that performs the 3-axis sample translations that are used to create the spatially resolved projections. (**B**) The manually operated cradle–gantry locking ring (yellow) is rotated to stretch an expansion ring over which the plinth adaptor (green) is mounted. (**C**) The animal cradle is positioned within the rectangular groove of the plinth adaptor and fixed in position using vertically oriented screws that attach to the plinth adaptor.

The PET/SPECT/CT system (Vector4CT, MILabs), as delivered, uses an expansion-ring-fitting coupling of its moving gantry support and cradle, which also automatically couples electrical services and gas tubes. Each cradle also features an identification chip that instructs the scanner software which cradle is installed such that spatial definition parameters are automatically set. The cabling and gas supplies for each cradle are run internally through the moving gantry. For the system described in this report, an adaptor plinth featuring an electronic identification chip is installed, but all electrical services and gas tubing are run externally rather than through the moving gantry. The cradle couples to this plinth using a rectangular-shaped cutting in the hexagonal profile of the cradle’s hexbase slider. Figure 3 shows a schematic of the PET/SPECT/CT loading apparatus.

### 2.6. Animal Cradles: Assembly and Peripherals

**Figure 4:**
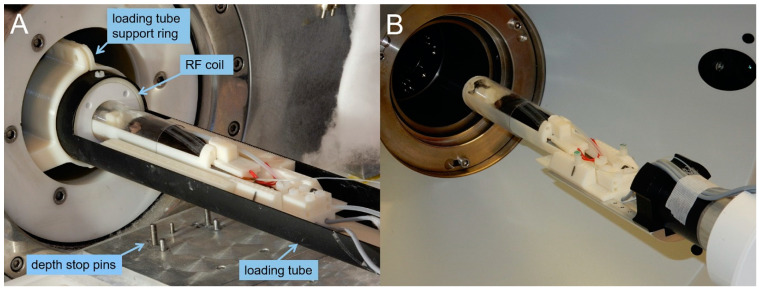
Use of the same animal cradle on the magnetic resonance imaging (MRI) and PET/SPECT/CT systems. (**A**) MRI scanner with loading tube, animal cradle and animal prior to imaging. (**B**) PET/SPECT/CT scanner with plinth adaptor, animal cradle and animal prior to imaging.

Photographs of the finished cradle assemblies for as used for MRI and PET/SPECT/CT imaging is shown in Figure 4. The animal cradles are produced from parts made using a commercial 3D plastic printer (F170, Stratasys, Birmingham, AL, USA) and are furnished for use with animal monitoring and maintenance equipment. Technical drawings were produced using commercial computer-aided design (CAD) software (Solidworks Professional 2018, Dassault Systèmes). The professional version of this software is used as drawings can be shared and modified by the mechanical workshop and others with whom we collaborate on part design.

**Figure 5:**
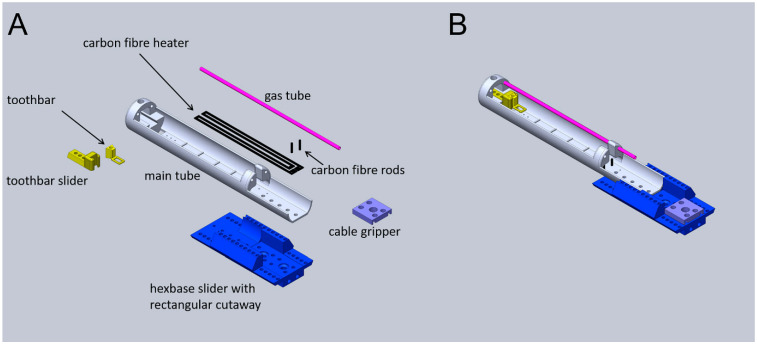
Schematic of the animal cradle as individual components (**A**) and assembled unit (**B**). The main tube (grey), where the animal lies, is permanently attached to the hexbase slider (blue). The toothbar features both horizontally and vertically adjustable positioning (yellow). The cable gripper (purple) is available for tethering any additional services that may be required, and multiple grippers can be stacked if multiple cables or tubes are required. The gas delivery tube (pink) is permanently mounted. The ridges on the flat base of the main tube are for attachment of the electrocardiogram (ECG) plate. The respiration monitor clips into the aperture at the hexbase slider end of the main tube, the latter also acting as the access point for the rigid rectal thermometer. The sheath tube (not shown) is a snug fit over the support rings that are located at the front and towards the back of the main tube. The carbon fibre heater, mounted underneath the main tube, is coupled to the power feed wire through the carbon fibre rods.

The cradle parts are joined using nylon screws (from an RS Pro 600 nylon screw kit, part number 524-095; RS Components, London, UK) with holes for screw thread tapping and clearance included at print time, and screw threads are manually tapped, where required. Most components feature an excess of both tapping and clearance holes for futureproofing. The cradle and its component parts are shown in Figure 5.

The main tube described in this report is produced at a 30.5 mm outer diameter such that standard, commercial thin wall tubing (part 09338, Visipak tubing, Sinclair and Rush, Rochester, UK) can be used as a sheath to contain the anaesthetic gas around the animal. Cradles having different diameters can be produced simply by changing one diameter in the CAD drawings of both the main tube and its hexbase slider. The diameters of the cradles we have used at our facility to date (23.5, 25.6, 30.5 mm) have been selected according to the sizes of the sheathing tubes we have available. Other sizes can be used, and sheaths can be adapted to any size by cutting a larger-diameter tube along its length and removing the excess material to form a smaller-diameter sheathing tube.

#### 2.6.1. Production of Heating Resistor

A 4-legged heater element, produced from 750-m-thick carbon fibre sheeting (part 764-8700; RS Components, UK) is coupled to a power feed wire (2-core screened cable, 3.1 mm o.d. part 749-2544; RS Components, UK) using 10-mm-long cuttings of 2-mm-diameter carbon fibre rods (CFROD-2-1; EasyComposites, Stoke on Trent, UK) using a compression fitting through a 2 mm tap hole in the carbon fibre heater element. The rod–supply cable junction is formed with a silver paint (part 1239911; RS Components, UK)-bonded connection positioned such that it can be positioned below the imaging plane of the Small-Animal Radiation Research Platform (SARRP) system, and the cable ends with a non-magnetic 15-pin D-sub connector (female F15S0G0-4212, male F15P0G0-4212, hood FMK2GNM; Toby Electronics, Banbury, UK) for attachment to the magnet room filter panel. For scanners operating a more conventional X-ray gantry rotation than the SARRP, the heater element can be coupled to the supply wire using metal screws, nuts and O-rings to form a very robust connection.

#### 2.6.2. Production of Gas Delivery Tube

Anaesthetic gas was delivered to the cradle using semi-rigid tubing (Portex™ Fine Bore LDPE Tubing, 30m, 2 mm ID, 3 mm OD; Smiths Medical) that featured a small nick hole positioned near the head. This delivered gas near to, but not face-on towards, the head to reduce any risk of flow-pressure-related respiratory depression. The tubing is terminated with a Luer-barb tube fitting (selected from a low-pressure systems fittings kit 7318220; Bio-Rad, Watford, UK) that attached to the scanners’ existing gas supply tube (which is also terminated with a Luer-barb fitting). The tube is passed through 2 close-fitting clearance holes in the main tube, and at the mouth bar end of the main tube, the gas delivery tubing is blocked with the shaft of an M3 nylon screw.

#### 2.6.3. Production of Graphene Piezo-Electric Respiration Monitor Device

**Figure 6:**
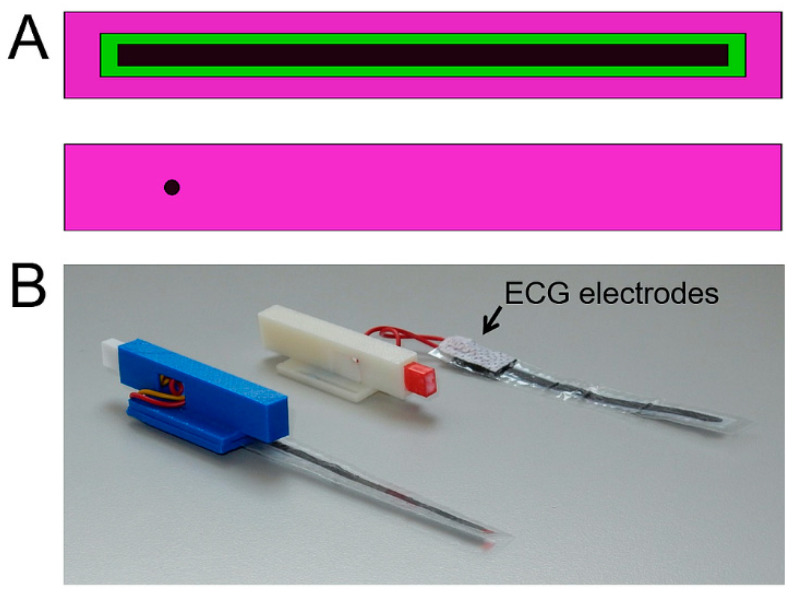
Assembly of the graphene-based piezo-electric monitor (GPERM). (**A**) Schematic of the internal surface of the laminated sheet showing the layering of the materials. The 6 × 90 mm^2^ polyvinylidene fluoride (PVDF) strip (green) is painted on both sides with a 3 × 85 mm^2^ graphene stripe (black). The graphene stripe is in direct contact with the 12 × 100 mm^2^, 2-layered lamination sheet (purple). The exteriorisation of the graphene stripe is realised through a 2 mm punch hole of the external surface of the laminated sheath to allow connection with the ECG pad. (**B**) Photograph of the laminated sheet with the ECG pads and the complete assembly. The latter is formed such that it clips into position in the animal cradle, and features height-adjustable feet to allow ease of use in a variety of cradle diameters.

Respiration was monitored using a graphene-based piezo-electric monitor (GPERM) formed from a strip of 110-m-thick, poled uniaxial polyvinylidene fluoride (PVDF) polymer (Precision Acoustics, Dorset, UK) that was cut into 6 × 90 mm^2^ long strips and onto which 3 × 85 mm^2^ stripes of particulate graphene (Elicarb, Thomas Swan, Durham, UK) were painted on each side. The area of painting was restricted using masking sheets prepared from envelope-label stickers into which 3 × 85 mm^2^ apertures were formed using a laser cutter (Speedy100, Trotech, Boldon, UK), and the painted strip was encased in thin, 75 g/m^2^, laminator sheet plastic, which had a 2-mm-diameter hole punched through for exteriorisation of the conductive graphene. CT-compatible electrocardiogram (ECG) pads (Neotech micro, Inspiration Healthcare) that feature carbon fibre wires were positioned over the punched holes to couple the graphene strip to the signal carrier wires that were cut to ca. 50 mm lengths, and the strip and cable were mounted into a 3D-printed cartridge that was terminated with an RCY connector (Socket part number 688-1448, plug part number 688-1445, female pins part number 688-1436 and male pins part number 161-3367 from Japanese Solderless Terminals) for coupling to its flying lead and non-magnetic 15-pin D-sub connector. A cartoon and a photograph of the assembly is shown in Figure 6.

#### 2.6.4. Production of ECG Detection Plate

**Figure 7:**
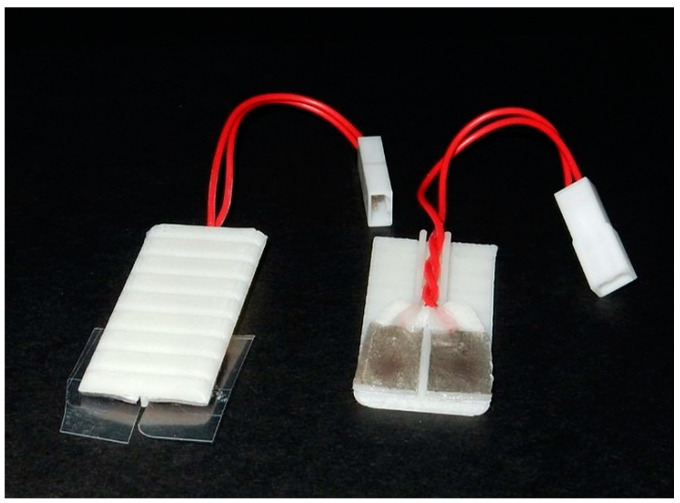
Assembly of the ECG detection plate. The indents on the lower surface allow the ECG detection plate to be clipped in place in the main animal cradle. The ECG pads are mounted onto the upper surface of the 3D-printed plate.

ECG was measured using the same CT-compatible ECG pads as for coupling the respiration monitor to its cabling. The pads were mounted on a 3D-printed plate that clips into position within the main tube. The carbon wires on the ECG pads are cut to approximately 50 mm in length and terminated with an RCY connector that is compression-fitted within a groove in the main tube when it is in use (see Figure 7). When not in use, the ECG plate is stored in a sealed vial that contains ca. 0.2 mL of water in it so as to keep the pads hydrated. A 2-core shielded signal carrier cable connects this RCY connector to a non-magnetic 15-pin D-sub connector for coupling at the MRI scan room filter panel. This cable passes through the rear of the magnet, suspended in the air, so it is not in direct contact with the gradient set (to minimise vibrations). In this arrangement, animal services run through both front and back ends of the magnet.

#### 2.6.5. Production of Rectal Thermometer Shaft

**Figure 8:**
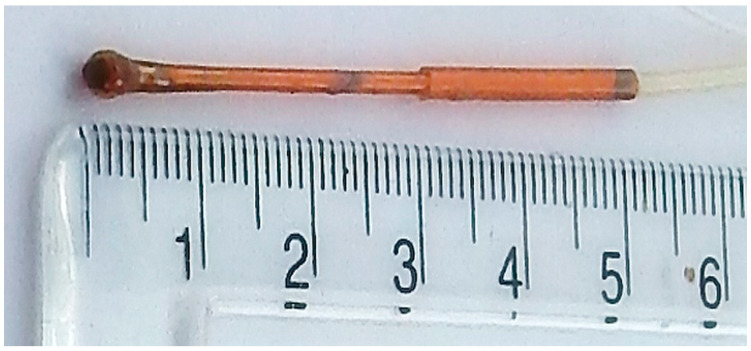
Close-up photographs of the fibre-optic thermometer probe.

A fibre-optic thermometry system (Accusens, Opsens, Canada) was used for monitoring the animals’ temperature, which was maintained using an imaging-compatible heating system described previously [9]. The blunt end of the thermometer probe was rounded, as depicted in Figure 8, using a crafted ball of epoxy glue so as to protect the animal from scratch-related injury.

With age, these balls will detach, but regular preventative maintenance checks have prevented this happening whilst in use, and repairs can be performed in under 30 min.

### 2.7. ECG and Respiration Signal Processing

For MRI, all animal service electrical connections are passed through a low-pass RF filter (series 700 high-performance filtered connector, 4000 pF capacitance Pi filter type, part number SCI 56-715-005; API Technology Corp., Milton Keynes, UK), which is mounted on a detachable plate on the scan room RF filter panel.

Physiological signal recording and display were performed using a standard commercial signal processing unit (MP150 base unit with UIM100C interface unit and DA100C (respiration) and ECGMRI100C (ECG) amplifier units). Analog respiration and ECG signals were passed to a custom-made gating control unit, which conditioned the signals and created TTL control signals to gate and trigger the relevant scans. The r-wave to ECG-gating control pulse signal propagation delay was <2.5 ms. The respiratory motion to respiratory gating control signal propagation delay was >50 ms as a rather harsh 10 Hz low-pass filter, incorporated in the DA100C amplifier, was used on this signal to eliminate 50 Hz mains noise. This delay was manageable as the MRI scan modes use dynamic reacquisition of data corrupted at entry to breath and the PET/SPECT/CT system bins data with the option of a propagation delay offset correction to the list mode data.

### 2.8. Transfer of Animals in the Cradle from One Scanner to Another

**Figure 9:**
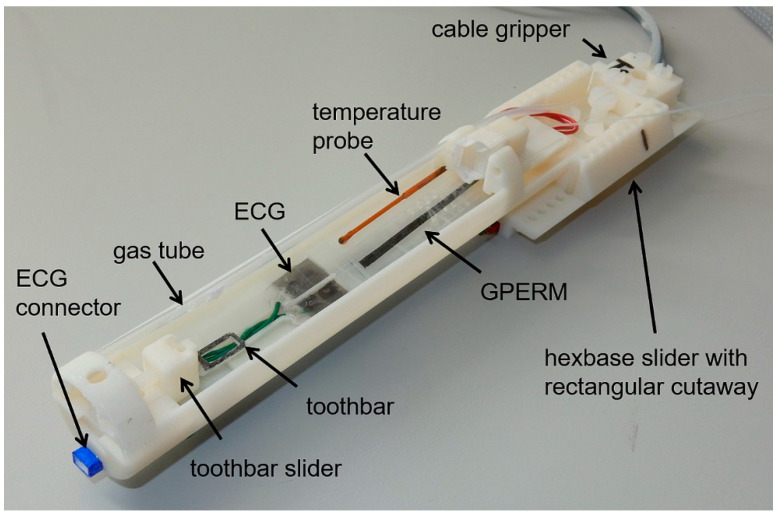
Photograph of the complete animal cradle assembly.

A photograph of the complete animal cradle assembly is shown in Figure 9. The following sequence was used to transfer the animal cradle assembly from the 7 T MRI scanner to the MILabs PET/SPECT/CT scanner. The loading tube was removed from the imaging isocentre to the operator position at the end of the scan (as in Figure 1. Removal of the cradle from the loading tube was performed in sequence: removal of the cradle from the imaging to the loading hexbase adaptor, disconnection of the ECG cable from its RCY connector, disconnection of the respiration monitor’s flying lead D-sub connector from the scan room filter panel, disconnection of the fibre-optic thermometer from its flying lead ferrule connector, disconnection of heating power flying lead D-sub connector from the scan room filter panel and, finally, disconnection of the anaesthetic gas supply. Where a cannula is present, it and its associated syringe are prepared for transport with the cradle. The cradle is then moved to the PET/SPECT/CT scanner, where the gas supply, heater power cable, respiration monitor, fibre-optic thermometer and ECG monitors are reconnected in that order. This order is chosen to minimise any risk of the animal waking up during transfer. Transfer from PET/SPECT/CT to MRI is the reverse process.

### 2.9. Demonstration of Multi-Modal Imaging

#### 2.9.1. MRI

MRI was performed on a 7 T, 210 mm VNMRS horizontal bore preclinical imaging system equipped with a 120 mm bore gradient insert (Varian Inc., Palo Alto, CA, USA). RF transmission and reception were performed using a 32-mm-diameter RF coil (Rapid Biomedical GmbH, Rimpar, Germany).

A B6SJLCD45-WT mouse was imaged using cardio-respiratory gated 3D spoiled gradient echo MRI. Structural imaging was performed using a cardio-respiratory gated 3D spoiled gradient echo scan with TR 2.52 ms, TE 1.08 ms, field of view (FOV) 64 × 32 × 32 mm^3^, matrix 256 × 128 × 128, gradient spoiling with 168 mT/m for 0.72 ms in all three axes, RF hard pulse duration 16 μs, flip angle (FA) 5° and RF spoiling. Data were acquired in blocks of 32 k-space lines per R-wave, and the two data blocks acquired prior to detection of each breath were reacquired immediately after the same breath [8]. A single average was acquired to give a 3D data set with 250 µm isotropic resolution in less than 2 min. Dynamic contrast-enhanced (DCE)-MRI was performed using a cardio-respiratory gated 3D spoiled gradient echo scan with TR 1.6 ms, TE 0.61 ms, FOV 64 × 32 × 32 mm^3^, matrix 128 × 64 × 64, gradient spoiling with 137 mT/m for 0.39 ms in all three axes, RF hard pulse duration 16 μs, FA 5° and RF spoiling. Data were acquired in blocks of 64 k-space lines per R-wave, and the two data blocks acquired prior to detection of each breath were reacquired immediately after the same breath [8]. Thirty repeats of the 3D scan were performed, with an average scan time of about 14 s each, with variations in the instantaneous breathing and heart rates resulting in variations in scan times. To account for this, the times of acquisition of the central k-space data were automatically recorded for each frame such that correct temporal indexing in the tracer distribution time domain could be performed. A contrast agent (30 μL Omniscan; GE Healthcare, Chalfont St Giles, UK) was infused in the lateral tail vein over 5 s at the start of frame 11/30.

#### 2.9.2. CT Imaging

CT was performed using the VECTor^4^CT system (MILabs, Utrecht, The Netherlands). Following MRI, the mouse was transferred in the cradle to the CT system. A cardio-respiratory gated CT scan was acquired: full 360° rotation, 1 bed position covering the whole mouse, X-ray tube settings of 50 kV and 0.24 mA, 1 degree per step, 32 projections per step, 2 × 2 binning and 20 ms exposure time. In addition, an anatomical reference image was acquired to allow easy localisation of radiotracer uptake and co-registration to the MRI acquisitions: ungated, full 360° rotation, 1 bed position covering the whole mouse, X-ray tube settings of 50 kV and 0.24 mA, continuous rotation at 40 degrees/s, 2 × 2 binning and 20 ms exposure time.

To demonstrate CT compatibility of the complete cradle apparatus, a post-mortem CT acquisition was performed: full 360° rotation, 2 bed positions covering the whole mouse, 0.75 degrees per step, 1 projection per step, 1 × 1 binning, X-ray tube settings of 55 kV and 0.19 mA and 75 ms exposure time.

All images were reconstructed using MILabs reconstruction software v11.0, applying a cone-beam filtered backprojection (Feldkamp algorithm). Beam hardening and ring artefacts were corrected, and the voxel numbers were converted into Hounsfield units by using a premeasured calibration factor. A 0.08 mm voxel grid was used to produce the post-mortem CT image. A 0.2 mm voxel grid was used for live imaging, together with 1 respiratory gating phase (window = 0.6, phase = 0.5) and 4 cardiac gating phases for the cardio-respiratory gated acquisition.

#### 2.9.3. SPECT Imaging

SPECT imaging of the abdomen, including the kidneys, was performed in the cradle on the same animal, during the same anaesthetic session and using anaesthesia and physiological maintenance, as described above. SPECT imaging was performed using the single-gantry VECTor^4^CT system fitted with the HE-UHR-RM PET/SPECT collimator (1.8 mm pinholes). CT imaging and SPECT imaging were performed immediately after MRI, with the mouse transferred in situ; ^99m^Tc-diethylene-triamine-pentaacetate (^99m^Tc-DTPA) was used as a contrast agent for SPECT. Data were acquired in list mode (0–1200 keV) using MILabs acquisition software v11.0. Triple-energy-window-based scatter correction was applied for the ^99m^Tc photon peak window (126.0–154.0 keV with background windows set to 120.4–126.0 keV and 154.0–159.6 keV). MILabs reconstruction software v11.0 was used to reconstruct the ^99m^Tc-DTPA image on a 0.6 mm isotropic 3D voxel grid using dual-matrix similarity-regulated ordered-subset expectation maximisation (dual-matrix SROSEM) [10], 1 respiratory gating phase and 4 cardiac gating phases. After reconstruction, the SPECT and the corresponding CT data were co-registered and resampled to equivalent 200 μm voxel sizes using the same software package. CT-based attenuation correction was applied. Reconstructed images were viewed and analysed using PMOD v.3.37 (PMOD Technologies, Zurich, Switzerland). ^99m^Tc-DTPA (34.9 MBq) was injected through a cannula into the lateral tail vein at the start of frame 3/30, and data were acquired for 15 min at 1 min post-injection (2 bed positions containing the top of the liver, kidneys and bladder; 30 frames of 30 s; 15s/bed position; 0–1200 keV). A CT image was acquired for anatomical referencing and to aid co-registration between CT, SPECT and MRI.

The transformation between MRI and PET/SPECT/CT image space was found by registering the average of the DCE-MRI image volume sequence to the CT image volume. A rigid multi-modal image registration algorithm (Matlab) was used to find the rigid-body transformation, and this was then used to initialise a non-rigid multi-modal registration [11]. The non-rigid registration is necessary because there is a non-linear spatial transformation between the MRI and CT coordinate systems. The registration tools can be found at https://ora.ox.ac.uk/objects/uuid:4ad2c11e-2cda-4e2e-b79c-8c7d31577101 with the DOI:10.5287/bodleian:xqOX58rrY (resolving to https://doi.org/10.5287/bodleian:xqOX58rrY).

## 3. Results and Discussion

This cradle system has been used successfully in thousands of scans by several groups over several years. It is currently installed, at our institute, on three MRI scanners (Bruker and Varian), PET/SPECT/CT (MILabs) and an image-guided radiotherapy system (SARRP) upon which MR image-guided radiotherapy is performed in abdominal tumours as a matter of routine [12]. Further modifications continue to be made to it, and this is made easy by the availability of spare mounting holes on most components and the rapid production of new components on the 3D printer.

**Figure 10:**
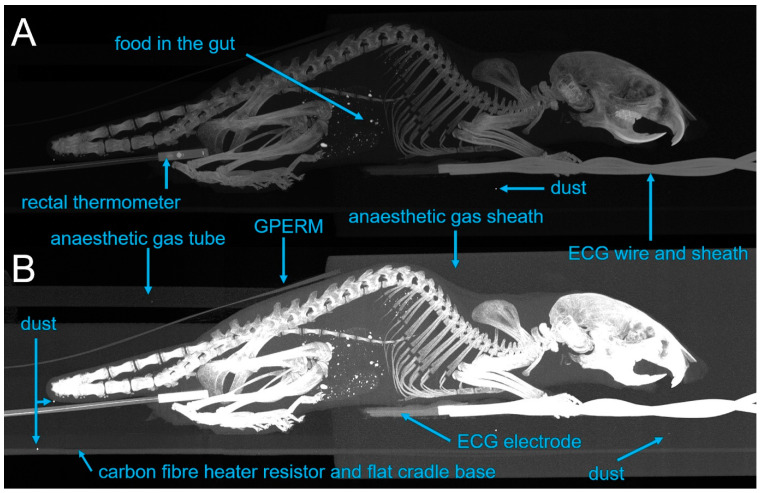
Maximum-intensity projection (MIP) CT images of the entire assembly using a standard (**A**) and a high-contrast-enhanced version (**B**) to highlight different features within the same MIP. Parts of the skeleton (unlabelled), GPERM strip, ECG electrodes, ECG cable sheath and the fibre-optic thermometer unit can be identified, as well as parts of the printed cradle. Bright dots are visible in the abdomen and are derived from the foodstuff. Additionally, three spots of unspecified material, presumably dust, can be seen.

A sagittal-view maximum-intensity projection (MIP) image of a mouse in the cradle with a fibre-optic thermometer and heating element, the GPERM and hydrogel ECG electrodes showed no unacceptably high signal intensities derived from the animal support apparatus, indicating its CT compatibility. MRI compatibility has been previously demonstrated, though not described in detail [9,13,14]. A sagittal-view MIP presentation of a CT image is shown in Figure 10.

None of the physiological monitoring devices are the brightest objects in the image and so will not present a significant source of image corruption through high levels of X-ray absorption and scattering.

The time required for transfer of the animal from the anaesthetic induction box to the scanner and the start of the first scanner operation is typically <60 s, whilst transfer of animals between scanners is below <90 s. These processes are very efficient, present a very low technical burden to the operator and are robust.

**Figure 11:**
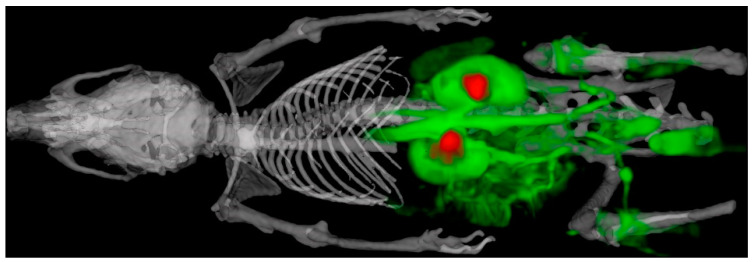
Multi-modal SPECT/CT/DCE-MRI of the mouse using the system-agnostic, adaptable and extensible animal support cradle system. The skeleton, kidneys and major vessels to the kidneys are marked up in the MIP. The skeleton (white) was imaged by CT, while ^99m^Tc-diethylene-triamine-pentaacetate (^99m^Tc-DTPA) (red) and gadodiamide (green) were used for SPECT and DCE-MRI of the kidneys, respectively.

A composite image formed from DCE-MRI, CT and ^99m^Tc-DTPA SPECT is shown in Figure 11. The ungated CT and cardio-respiratory gated MR images were used to drive both the rigid- and non-rigid-body registrations. The unprocessed CT, MRI and SPECT images can be found at https://ora.ox.ac.uk/objects/uuid:4ad2c11e-2cda-4e2e-b79c-8c7d31577101 with the DOI:10.5287/bodleian:xqOX58rrY (resolving to https://doi.org/10.5287/bodleian:xqOX58rrY).

Multi-modal SPECT/CT/DCE-MRI was performed, and images could be easily co-registered, displayed, analysed and presented. The renal pelvis and calyxes, as rendered from the SPECT image following renal excretion of ^99m^Tc-DTPA, overlaid well with the kidneys, as rendered from DCE-MRI. No image distortions attributable to material selection were observed, the animal handling apparatus was easily transferrable between MRI and CT/SPECT and cardio-respiratory signals allowed for prospective gating and triggering for MRI and CT/SPECT, respectively.

Figure 11 shows a tri-modal, cardio-respiratory-synchronised image produced using scanners from two different manufacturers, from the same animal positioned in the same cradle and all acquired within 5 min of each other. The experiment time defined as from induction to full recovery of anaesthesia was 70 min for a single animal. However, throughput can be increased significantly by using two cradles simultaneously and starting the next animal on MRI whilst the previous animal is transferred to SPECT/CT, similar to the workflow described for MRI-guided radiotherapy [12]. This high throughput is enabled, in significant part, through the use of well-designed animal handling cradles that are both easy to produce in bulk and easy to use. The physiological monitoring is minimally invasive, and excellent-quality ECG and respiration signals result. Though only skin contact is used to detect the ECG, the signals are sufficiently strong and insensitive to the imaging gradients to allow prospective gating control. As a result, the speed advantages described previously [6,8,13] are enabled without the use of invasive needle positioning and whilst avoiding the presence of metal in the CT imaging FOV. The respiration signals are produced through gentle contact of a flexible sheet with the animal, with no requirement to apply pressure to the body as so often required when using pneumatic pressure balloons, and so, there is no need to invoke a postural defect.

The cradles are quick and very simple to assemble; printing of a cradle main tube, hexbase base slider, cable grippers and tooth bar components takes approximately 6 h using our F170 printer. Removal of space-filler material in a solvent bath takes a further 6 h, and post-bath drying takes <3 h. Physical assembly of the prepared components takes <1 h. It is, therefore, quite possible to produce a new cradle from scratch within one working day of demanding it.

The cradle main tube has a flat base with a variable depth and height tooth bar, making it very easy to position the animals with repeatable prone posture. The underside of the flat base also allows the sheet resistor to be mounted very easily and to give a good quality of heating, as described previously [9]. The upper side of the flat base has a series of ridges spaced by 20 mm that allow a variety of devices, including the ECG plate, to be clipped into a variety of positions along the length of the cradle. Good thermal contact of animal to cradle is achieved, and a range of animal sizes can be accommodated, even when ECG signals are required.

Production of GPERMs is a simple modification of a design described previously [15] and requires approximately 1 h of printing time, 1 h of solvent bath time and 1 h of drying time. Once all the required parts are available, the assembly of a prepared piezo-electric strip into the housing takes <10 min. Preparation of the piezo-electric strip requires a series of straightforward though intricate and sequential procedures, and so strips are prepared in batches of five or more not individually. A jig, also prepared on the 3D printer, was used to cut the PDVF sheet into 6-mm-wide strips whilst protecting the operator’s fingers from the sharp blade used. Masks for painting the graphene strips were prepared using envelope-label stickers that were cut using a laser cutter, and the laminator sheets were cut into strips again using a jig similar to that used for cutting the PVDF sheet. The thin layer of graphene, painted on a fluorocarbon polymer, is compatible with both MRI and CT (and by inference PET and SPECT) and did not result in image distortions (Figure 10). The detector is thin, flexible and sensitive, generating respiration signals robustly and without the need to apply pressure to the body with the consequent postural distortion. As described, the respiration monitor provides a rather global measurement of respiration and so may not be optimised for any specific organ that is subject to respiratory motions. A simple redesign of the pad using, for example, a smaller area of painted graphene could be used to optimise the measurement of the breathing motions locally. The design of the entire apparatus allowed easy and quick transfer of the animal handling apparatus between different imaging systems through the use of quick-fit adaptors.

Production of the ECG plates was very fast; each base takes approximately 10 min to print. Solvent stripping and drying takes a further 60 min, but the plates are small enough that they can often be produced in bulk during the print runs for other objects. Once all the required parts are available, the assembly of an ECG plate takes <10 min. The units were formed by shortening the commercial pads’ carbon wires, which were spiralled into a twisted pair in order to minimise pickup of gradient-switching-induced noise in MRI and were attached to quick-fit connectors. The pads were mounted on a support plate, which clips in place onto the ribs of the cradle base, making it easy to adjust its position in relation to the animal. These ECG pads allowed generation of robust ECG signals, even in the presence of high-demand gradient switching during MRI. The sheaths of these wires were relatively bright in CT but no brighter than many of the bones and so do not create any additional confounding source of streak artefact [9]. In this report, we describe their use in contact with the shaved chest, but they can be expected to work wherever contact with the skin can be achieved.

A set of apparatuses has been described that enables multi-modal imaging to be performed using scanner equipment from multiple vendors, each of which is designed without reference to any other. This allows the advantages of each technique to be exploited, whilst maintaining the ability to match organs on a pixel-wise basis and to provide a multi-parametric characterisation of anatomy, physiology and pathology. This provides clear opportunities for improvements in both scientific content and animal welfare as better data can be produced from fewer animals, especially when paired statistics that can be applied across both the time (repeated imaging) and image modality dimensions are used. In some cases, e.g., SPECT-CT, PET-CT and PET-MRI, multi-modal imaging can be performed within a single scanner assembly, in which case the issues of cradle transfer are largely irrelevant. However, in most cases, separate MRI, PET and SPECT systems would be used, and a SPECT-MRI system does not yet exist. As such, the cradle system described (and any variation on a theme) provides an opportunity to standardise animal handling procedures in a range of environments (scanners) and achieve improved performance, so aiming for maximum reproducibility [16]. Whilst there has been significant effort in defining how well scanners should operate [17] and how in vivo experimentation should be practised [18,19], there is little detail on how to achieve optimal scanner performance with best practises in animal welfare. The need for immobilisation of the animal through the use of general anaesthesia is recognised [20,21], but methods for maintaining temperature and monitoring physiology across a range of multi-modal, multi-vendor imaging systems are not well described. Through a combination of careful material and device selection, we have described an approach that allows animals to be transferred whilst under general anaesthesia between any of the tomographic scanners we currently or have previously operated (Bruker and Varian MRI, MI-Labs PET/SPECT/CT, Xstrahl SARRP radiotherapy and Siemens Inveon PET-CT). Extension to other systems from other suppliers is expected to be straightforward. The heaters, ECG and respiration monitors are easy to use and compatible across all of the cradle sizes we use (23–39 mm diameter). The smallest cradles are designed to allow use with a 26 mm internal diameter, 100 mm long, whole-body RF coil for MRI [8] and with the high-resolution, small-bore mouse collimators on the MILabs PET/SPECT/CT system.

## 4. Conclusions

In summary, we believe this is the first report of a comprehensive, yet adaptable, animal handling system that is compatible across the modalities most often used in tomographic imaging (PET, SPECT, CT and MRI) and that allows the use of the highest CT magnification factors and the smallest bore collimators, RF and gradient coils, thereby offering the best image quality per system. The set-up is minimally invasive, cheap and easy to implement and for multi-modal, multi-vendor imaging of small animals.

## Abbreviations

MRI	magnetic resonance imaging
PET	positron emission tomography
SPECT	Single-photon emission computed tomography
CT	computed tomography
TTL	transistor–transistor logic
v/v	volume to volume percent
RF	radiofrequency
hexbase	hexagonally shaped dovetail
2D/3D	2/3 dimensional
SARRP	Small-Animal Radiation Research Platform
CAD	computer-aided design
ECG	electrocardiogram
FOV	field of view
GPERM	graphene piezo-electric respiration monitor
PVDF	polyvinylidene fluoride
VNMRS	Varian nuclear magnetic resonance system
FSE	fast spin echo
bSSFP	balanced steady-state free precession
FLASH	fast low-angle shot
DCE-MRI	dynamic contrast-enhanced MRI
HE-UHR-RM	high-energy, ultra-high-resolution, rat mouse
MIP	maximum-intensity projection
